# Flexible Tricolor Flag-liked Microribbons Array with Enhanced Conductive Anisotropy and Multifunctionality

**DOI:** 10.1038/srep14583

**Published:** 2015-09-28

**Authors:** Qianli Ma, Wensheng Yu, Xiangting Dong, Ming Yang, Jinxian Wang, Guixia Liu

**Affiliations:** 1 Key Laboratory of Applied Chemistry and Nanotechnology at Universities of Jilin Province, Changchun University of Science and Technology, Changchun 130022.

## Abstract

Anisotropically conductive materials are important components in subminiature devices. However, at this stage, some defects have limited practical applications of them, especially low anisotropic degree and high cost. Here, we report novel tricolor flag-liked microribbons array prepared by electrospinning technique. The tricolor flag-liked microribbons array is composed of parallel microribbons, and each microribbon consists of three different regions, just like tricolor flag. The tricolor flag-liked microribbons array is only electrically conductive in the direction parallel to the microribbons, whereas in the perpendicular and thickness directions are insulative. The electrical conductivity along parallel direction reaches up to 8 orders of magnitude higher than that along perpendicular direction. The degree of anisotropy in present study is increased by 2 orders of magnitude than that of the anisotropically conductive material in references reported before. Besides, other functions can be conveniently assembled into tricolor flag-liked microribbons array to realize multifunctionality. Owing to the high electrical anisotropy and multifunctionality, tricolor flag-liked microribbons array will have important applications. Furthermore, a universal technique to prepare microribbons with three functional regions has been established for fabricating excellent multifunctional materials.

Anisotropically conductive materials have been widely investigated as one kind of green materials in electronic packaging owing to their many advantages, such as lead-free and environmentally friendly, high density connection in narrow space achievable, and insulative in needless directions so that the short circuit can be prevented. Generally, anisotropically conductive materials are films which have different conductivities in different directions^1^,^2^,^3^,^4^,^5^,^6^. In particular, the researchers have spent a great deal of efforts to achieve the anisotropically conductive materials which are conductive along one direction of their surfaces and insulative along the others. Typically, anisotropically conductive materials are single or multilayer thin film materials that comprehend parallel-arranged one-dimensional (1D) conductive units, such as molecular chains or conductive fibers, and these 1D conductive units are separated from each other with air and/or interfaces (act as the resistors)^7^,^8^,^9^. Thus, the electrical conduction in the direction parallel to the 1D conductive units is higher than that in the perpendicular direction due to different electrical connection in the two directions. Up to now, some kinds of anisotropically conductive materials have been fabricated. Jeon, *et al.*^10^ studied the optical and electrical properties of preferentially anisotropic single-walled carbon-nanotube films. The carbon nanotube network is composed of metallic and semiconducting nanotubes embedded in an air dielectric host, and the electrical properties are different along parallel and perpendicular directions. Ra, *et al.*^11^ fabricated the multiwalled carbon nanotubes (MWCNTs) embedded polyacrylonitrile nanofiber paper via electrospinning, and its conductivity along parallel direction was three times higher than that along perpendicular direction. Majewski, *et al.*^12^ achieved anisotropic ionic conductivity in block copolymer membranes by magnetic field alignment. The conductivity along parallel direction of the membranes reached up to 1000 times higher than perpendicular direction. In year 2014, Huang, *et al.*^13^ fabricated an anisotropic conductive polymer film by shear-flow inducing MWCNTs to assemble into well-ordered parallel MWCNT stripes vertical to the shear flow. The electrical measurement showed that the electrical resistivity along the MWCNT stripes was 6 orders of magnitude lower than that orthogonal to the stripes, which was the reported most striking conductive anisotropy for the plastic anisotropic conductive materials. Very recently, Liu, *et al.*^14^ prepared highly aligned polyimide composite fibers film with graphene nanoribbon and carbon nanotube hybrids as nanofillers by means of electrospinning. The conductivity ratio between the parallel and perpendicular directions of the product also reached up to 6 orders of magnitude. On the basis of many literatures concerning anisotropically conductive materials, it can be noticed that how to effectively enhance the degree of anisotropy of anisotropically conductive materials is an important and urgent issue.

Electrospinning technique has been widely adopted to effectively fabricate 1D nano/micro materials^15^,^16^,^17^,^18^,^19^,^20^,^21^,^22^,^23^. The electrospun products have some unique advantages, such as long length (up to several meters), good flexibility (owing to the polymer template) and low power consumption. Furthermore, the order of the electrospun products can be arranged by using special collector^24^,^25^,^26^,^27^. In this work, we present a simple one-pot electrospinning method to acquire a new kind of anisotropically conductive material by using a specially designed spinneret. Our design concept for the novel anisotropically conductive material is to introduce two insulative regions on both sides of conductive region of a microribbon, and arrange the microribbons into parallel structure. The novel microribbons with three different regions were just like tricolor flag, and thus, we named the microribbons as tricolor flag-liked microribbons. Based on this structure, the conducting substance (PANI) can be effectively isolated by insulative regions in perpendicular direction, and thus, it is expected that very high conductive anisotropy can be achieved. Moreover, since multifunctionality of future materials is the development trend, establishing a universal method to assemble different components with various functions together becomes an meaningful subject of study. For the tricolor flag-liked microribbon, other non-conductive functional materials can be introduced into the two insulative regions to achieve versatility. In this work, we chose magnetic and photoluminescent materials as examples and respectively added them into the two insulative regions. Thus, the final tricolor flag-liked microribbons array not only possesses anisotropic conduction, but also photoluminescent and magnetic properties. Furthermore, it is feasible to replace these materials with other functional materials according to the application requirements to realize multifunctionality. From the viewpoint of multifunctional materials, the design concept and construction technique are of great significance to fabricate multifunctional micro/nano materials.

## Materials and Methods

### Chemicals

Methylmethacrylate (MMA), benzoylperoxide (BPO), Tb_4_O_7_, benzoic acid (BA), 1,10-phenanthroline (phen), FeCl_3_·6H_2_O, FeSO_4_·7H_2_O, NH_4_NO_3_, polyethylene glycol (PEG, Mr ≈ 20000), ammonia, oleic acid (OA), aniline (ANI), (IS)-(+)-camphor-10 sulfonic acid (CSA), ammonium persulfate (APS), anhydrous ethanol, CHCl_3_, DMF, and deionized water were used. All the reagents were of analytical grade and were directly used as received without further purification. The purity of Tb_4_O_7_ was 99.99%. The deionized water was homemade.

### Preparation of oleic acid modified Fe_3_O_4_ nanoparticles, Tb(BA)_3_phen complexes and PMMA

Fe_3_O_4_ nanoparticles with the saturation magnetization of 62.02 emu g^−1^ were obtained by using a facile coprecipitation synthetic method (see Electronic supplementary information)^28^. To improve the monodispersity, stability, and solubility of Fe_3_O_4_ nanoparticles in the spinning solution, the as-prepared Fe_3_O_4_ nanoparticles were coated with oleic acid as below: 1.5 g of the as-prepared Fe_3_O_4_ nanoparticles were ultrasonically dispersed in 50 mL of deionized water for 20 min. The suspension was heated to 80 °C under an argon atmosphere with vigorous mechanical stirring for 30 min and then 0.5 mL of oleic acid was slowly added. The reaction was stopped after heating and stirring the mixture for 40 min. The precipitates were collected from the solution by magnetic separation, washed with ethyl alcohol for three times, and then dried in an electric vacuum oven at 60 °C for 6 h. Then, all of the products were used to prepare spinning solution III. Tb(BA)_3_phen powders were synthesized according to the traditional method as described in the literature^29^. PMMA used in this study was prepared by oxidative polymerization of MMA according to the reference 30.

### Preparation of spinning solutions

Three different spinning solutions, respectively marked as I, II and III, were used to fabricate tricolor flag-liked microribbons array. The spinning solution I for the photoluminescent-insulative region consisted of Tb(BA)_3_phen, PMMA, CHCl_3_, and DMF. It has been known through our previous work that the optimum ratio of Tb(BA)_3_phen to PMMA is 1:10^28^. Thus for the preparation of the spinning solution I, 0.5000 g of PMMA and 0.0500 g of Tb(BA)_3_phen were added into the mixed solution of 9.3750 g of CHCl_3_ and 0.6250 g of DMF under magnetic stirring for 48 h.

As for the preparation of spinning solution II for the conductive region, 0.5000 g of PMMA was dissolved into the mixed solution of 9.3750 g of CHCl_3_ and 0.6250 g of DMF under magnetic stirring for 48 h. The mixture was then cooled down to 0 °C in an ice-bath. Then 0.1500 g of ANI and 0.1873 g of CSA were added into the above mixture and kept stirring for 2 h, followed by introducing 0.3676 g of APS for 30 min. The final mixture was allowed to react at 0 °C for 24 h. The impact of different amounts of ANI, CSA and APS on the conductivity of conductive region was discussed in Electronic supplementary information.

The spinning solution III for the magnetic-insulative region was composed of all the as-prepared oleic acid modified Fe_3_O_4_ nanoparticles, 0.5000 g of PMMA, 9.3750 g of CHCl_3_ and 0.6250 g of DMF. The mixture was firstly ultrasonically dispersed for 20 min and then stirred under mechanical agitation for 48 h.

### Fabrication of tricolor flag-liked microribbons array

**Figure 1:**
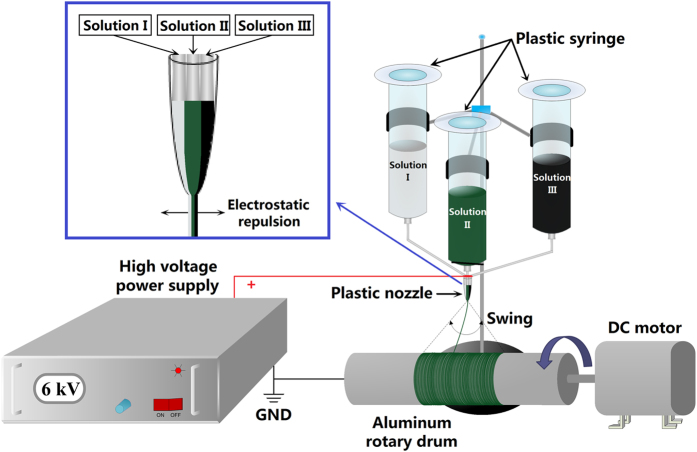
Schematic diagram of electrospinning equipment for preparing tricolor flag-liked microribbons array.

A sketch of the modified electrospinning setup is illustrated in Fig. 1, and more detailed manufacture process and utilization of the specially designed spinneret are provided in Electronic supplementary information. In the electrospinning process, three different spinning solutions respectively marked as I, II and III were loaded into three syringes. When the three spinning solutions flowed down to the plastic nozzle, they would form a triple parallel structure. During this process, the three spinning solutions would not blend owing to their viscous resistance. Subsequently, the three spinning solutions were stretched by electric field force to form a jet with triple parallel structure. The jet was then stretched to ribbon shape by electrostatic repulsion and solidified with the volatilization of solvents, thus a microribbon with three different regions was obtained. In order to obtain tricolor flag-liked microribbons array, an aluminum rotary drum was used as a collector, which was put about 18 cm away from the tip of the plastic nozzle. The aluminum rotary drum was 20 cm in length, 7 cm in diameter, fixed in horizontal, and the rotation speed was 1500 r min^−1^. A positive direct current (DC) voltage of 6 kV was applied between the spinneret and the collector to generate stable, continuous PMMA-based tricolor flag-liked microribbons array at room temperature of 20–22 °C and the relative humidity of 20–30%. Because the microribbon swung in the air due to the instability of electrospinning process, an array with a certain width could be collected on the surface of the aluminum rotary drum.

### Characterization

The phase compositions were identified by an X-ray powder diffractometer (Bruker, D8 FOCUS) with CuKα radiation. The operation voltage and current were kept at 40 kV and 20 mA, respectively. The morphologies and internal structures were observed by a field-emission scanning electron microscope (FESEM, XL-30) and a biological microscope (BM, CVM500E), respectively. The elemental analysis was performed by an energy-dispersive spectrometer (Oxford Instruments) attached to the FESEM. The electrical properties were measured by a Hall effect measurement system (ECOPIA HMS-3000) and a dielectric spectrometer (Concept 80). The fluorescent properties were investigated by Hitachi fluorescence spectrophotometer F-7000. The UV-Vis absorption spectra were recorded by a UV-Vis spectrophotometer (SHIMADZU UV mini 1240). Then, the magnetic performances were measured by a vibrating sample magnetometer (VSM, MPMS SQUID XL). All the determinations were performed at room temperature.

## Results and Discussion

### Morphology and internal structure

**Figure 2:**
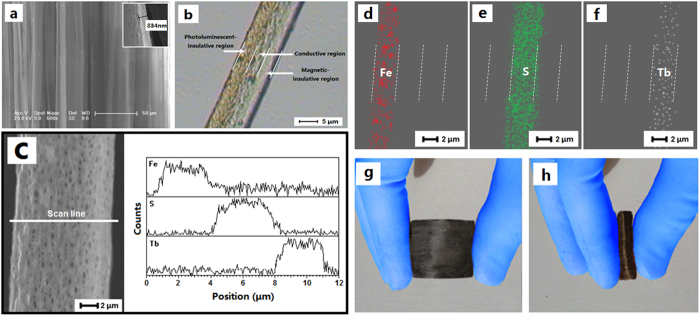
(**a**) SEM image of tricolor flag-liked microribbons array, the inset shows the thickness of a tricolor flag-liked microribbon; (**b**) optical microscope photograph of a single tricolor flag-liked microribbon; (**c**) SEM image and EDS line-scan analysis of a single tricolor flag-liked microribbon; (**d–f**) EDS mapping of a single tricolor flag-liked microribbon; and digital camera photos of the resulting tricolor flag-liked microribbons array: (**g**) an unbent tricolor flag-liked microribbons array; (**h**) bent tricolor flag-liked microribbons array.

Figure 2a displays a typical SEM image of the product. All the microribbons are aligned in the same direction, and the width of every single microribbon is *ca*. 10 μm and the thickness is less than 1 μm. Moreover, it can be seen that almost all the broadsides of these microribbons are facing up, with few exceptions, which is probably because this arrangement would help the microribbons lay in a more stable and lower potential energy state onto the aluminum rotary drum. The faced up orientation of broadsides for the microribbons can facilitate the isolation effect of insulative regions on the conductive region in perpendicular direction. Depending on the transmission light of optical microscope, the inner structures of the tricolor flag-liked microribbons can be observed. As revealed in Fig. 2b, a clear triple parallel structure, just like tricolor flag, can be seen in a microribbon: one side contains large quantities of tawny oleic acid modified Fe_3_O_4_ nanoparticles, the middle part is scattered with green PANI, and another side is transparent. In order to further determine the structure of each single tricolor flag-liked microribbon, energy dispersive spectrum (EDS) in line scan mode was performed, where Fe, S and Tb elements represent Fe_3_O_4_, PANI and Tb(BA)_3_phen, respectively. As shown in Fig. 2c, elemental Fe, S and Tb respectively exist in one side, middle and another side part of a tricolor flag-liked microribbon. The EDS mapping tests shown in Fig. 2d–f also manifest the same result.

From the above results, we can safely conclude that a novel material with a new and interesting structure has been successfully fabricated. Figure 2g, h present the typical digital camera photos of the resulting tricolor flag-liked microribbons array. One can see that the tricolor flag-liked microribbons array can be freely bent and folded. This is critically important for the application of the material in the flexible device.

### Electrical characteristics of tricolor flag-liked microribbons array

**Figure 3:**
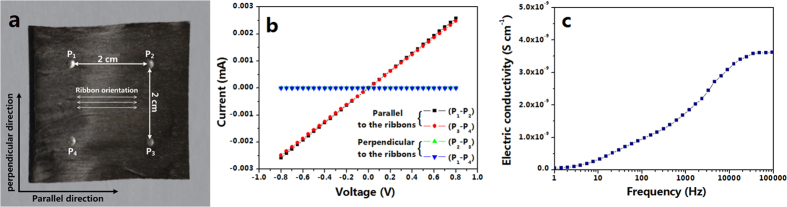
(**a**) Arrangement of the four contact points on a piece of tricolor flag-liked microribbons array; (**b**) *I–V* curves of the tricolor flag-liked microribbons array in the directions between P_1_ and P_2_, P_3_ and P_4_, P_2_ and P_3_, P_1_ and P_4_; and (**c**) variation of conductivity in the thickness direction of tricolor flag-liked microribbons array with alternating current frequency.

In order to investigate the anisotropic conductivity of the tricolor flag-liked microribbons array, a small piece of soldering tin was cut into four little pieces with diameter of *ca.* 2 mm and was pressed onto the sample (The sample has been cut into the size of *ca.* 3 cm × 3 cm × 0.5 mm), as indicated in Fig. 3a. Then the four pins of a Hall effect measurement system were respectively pressed on the four pieces of soldering tin. The relationships of voltage and current between P_1_ and P_2_, P_3_ and P_4_, P_2_ and P_3_, P_1_ and P_4_ were recorded by Hall effect measurement system, and the results were presented in Fig. 3b. The determined electrical conductivity between P_1_ and P_2_, P_3_ and P_4_, P_2_ and P_3_, P_1_ and P_4_ are listed in Table 1. One can see that the conductivity along parallel direction (P_1_–P_2_; P_3_–P_4_) is *ca.* 8 orders of magnitude higher than that along perpendicular direction (P_2_–P_3_; P_1_–P_4_), demonstrating that the tricolor flag-liked microribbons array has a strong anisotropy in conductivity. To the best of our knowledge, up to now, this value of conductivity ratio is the highest for anisotropically conductive materials.

The conductivity in the thickness direction of tricolor flag-liked microribbons array is also measured, by using a Concept 80 dielectric spectrometer. As seen in Fig. 3c, the measured conductivities are less than 4 × 10^−9^ S cm^−1^ in an alternating current frequency range of 1 Hz to 100000 Hz, indicating that the tricolor flag-liked microribbons array is insulative in its thickness direction. From the above measurement results, we can conclude that the tricolor flag-liked microribbons array is only electrically conductive in the direction parallel to the ribbons, whereas in the perpendicular and thickness directions are insulative.

**Figure 4:**
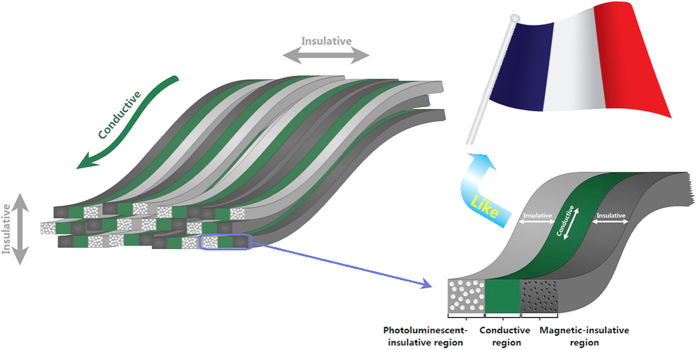
Schematic diagram of the property of tricolor flag-liked microribbons array.

As described in some literatures^8^,^11^, anisotropic conductivity of an electrospun fibrous film can be attributed to the different electrical connection between parallel and perpendicular direction. Electrons can move without block through the fibers in parallel direction, whereas a lot of interfaces among the fibers hinder the movement of electrons to some extent in perpendicular direction. However, as previously mentioned, the conductivity ratios between parallel and perpendicular direction of the most of products reported in literatures are very low, meaning that the interfaces can not effectively hinder the movement of electrons in perpendicular direction. The schematic diagram of tricolor flag-liked microribbons array is shown Fig. 4, in which the left part reveals a multilayered structure, and the structure of a single tricolor flag-liked microribbon is also proposed, which has interesting structure, just like tricolor flag (we use a picture of French flag to illustrate). Regarding the tricolor flag-liked microribbons array, because the conductive region of each tricolor flag-liked microribbon is sandwiched between two insulative regions, and almost all the broadsides of the flag-liked microribbons are facing up, the conductive regions of the tricolor flag-liked microribbons are separated by the plenty of insulative regions in perpendicular direction, which further blocks the movement of electrons. As for the thickness direction, because the electrospun microribbons are landed randomly onto the aluminum rotary drum, the conductive region in a microribbon has little chance to align with the other conductive region in the lower layer. Once adequate number of layers is collected, statistically, the conductive regions are misaligned and not connective in thickness direction, and thus, the tricolor flag-liked microribbons array is insulated in its thickness direction.

### Multifunctionalization by introducing photoluminescence and magnetism

**Figure 5:**
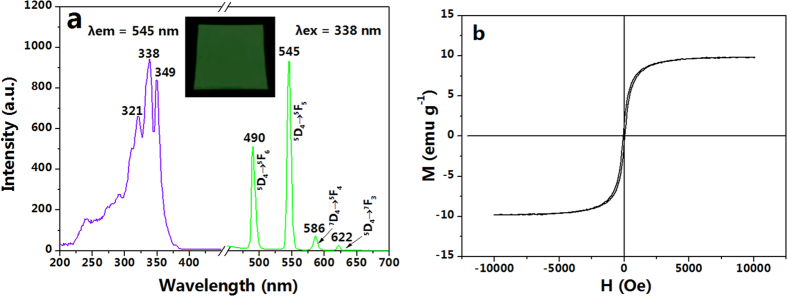
(**a**) Photoluminescence spectra of tricolor flag-liked microribbons array, the inset shows the digital photo of tricolor flag-liked microribbons array taken under the excitation of 338-nm ultraviolet light in the darkness; and (**b**) hysteresis loop for tricolor flag-liked microribbons array.

Figure 5a reveals the photoluminescence spectra of tricolor flag-liked microribbons array. Characteristic emission peaks of Tb^3+^ are observed under the excitation of 338-nm ultraviolet light and ascribed to the energy levels transitions of ^5^D_4_→^5^F_6_ (490 nm), ^5^D_4_→^5^F_5_ (545 nm), ^7^D_4_→^5^F_4_ (586 nm), and ^5^D_4_→^7^F_3_ (622 nm), and the ^5^D_4_→^5^F_5_ hypersensitive transition at 545 nm is the predominant emission peak. As seen in the inset of Fig. 5a, tricolor flag-liked microribbons array can emit obvious green fluorescence under the excitation of 338-nm ultraviolet light.

It is well known that the saturation magnetization of a magnetic composite material depends on the mass percentage of the magnetic substance in the magnetic composite material^28^,^29^,^30^. The saturation magnetization of the Fe_3_O_4_ nanoparticles before coating by oleic acid is 62.02 emu g^−1^, and the remanence nears zero (see Supplementary Figure S3). By using of the as-prepared Fe_3_O_4_ nanoparticles, the tricolor flag-liked microribbons array shows its saturation magnetization of 9.80 emu g^−1^ and remanence of zero, as seen in Fig. 5b. The magnetic properties indicate that the tricolor flag-liked microribbons array possesses superparamagnetic property.

The above analyses reveal that other functions, such as magnetism and photoluminescence, can be introduced into the insulative regions to realize multifunctionality of the tricolor flag-liked microribbons array, as long as the adopted materials do not compromise insulativity.

## Conclusions

We have fabricated a new type of anisotropically conductive material which is named as tricolor flag-liked microribbons array via simple electrospinning technology. The obtained tricolor flag-liked microribbons array consists of parallel-arranged tricolor flag-liked microribbons, and each tricolor flag-liked microribbon is composed of three different regions, which are photoluminescent-insulative region, conductive region and magnetic-insulative region, respectively. The conductive region is sandwiched between photoluminescent-insulative region and magnetic-insulative region. Owing to this unique structure, the electrical conductivity along parallel direction is more than 8 orders of magnitude higher than that along perpendicular direction, and the thickness direction of the product is determined to be insulative. Meanwhile, we have demonstrated that other functions, such as magnetism and photoluminescence, can be assembled into the insulative regions to realize multifunctionality of the tricolor flag-liked microribbons array, as long as the adopted materials do not harm insulativity. More importantly, the design concept and fabrication technique for the tricolor flag-liked microribbons array provide a new and facile approach for preparing other anisotropic materials with multifunctionality.

## Additional Information

**How to cite this article**: Ma, Q. *et al.* Flexible Tricolor Flag-liked Microribbons Array with Enhanced Conductive Anisotropy and Multifunctionality. *Sci. Rep.* **5**, 14583; doi: 10.1038/srep14583 (2015).

## Supplementary Material

Supplementary Information

## Figures and Tables

**Table 1:** Conductivity between P_1_ and P_2_, P_3_ and P_4_, P_2_ and P_3_, P_1_ and P_4_.

Directions	Conductivity (S cm^−1^)
P_1_–P_2_	(6.45 ± 0.03) × 10^−4^
P_3_–P_4_	(6.20 ± 0.02) × 10^−4^
P_2_–P_3_	(1.21 ± 0.01) × 10^−12^
P_1_–P_4_	(1.35 ± 0.02) × 10^−12^
