# Supplementary material for: Flexible Tricolor Flag-liked Microribbons Array with Enhanced Conductive Anisotropy and Multifunctionality
Source: Sci Rep. 2015 Sep 28;5:14583. doi: 10.1038/srep14583 (PMC4585964; doi:10.1038/srep14583)
Supplement: Supplementary Information [file srep14583-s1.doc]

**Flexible Tricolor Flag-liked Microribbons Array with Enhanced Conductive Anisotropy and Multifunction**

Qianli Ma, Wensheng Yu, Xiangting Dong,* Ming Yang, Jinxian Wang and Guixia Liu

Key Laboratory of Applied Chemistry and Nanotechnology at Universities of Jilin Province, Changchun University of Science and Technology, Changchun 130022.

[*] Prof Xiangting Dong

Key Laboratory of Applied Chemistry and Nanotechnology at Universities of Jilin Province, Changchun University of Science and Technology, Changchun 130022.

Tel.: +86-0431-85582574

Fax: +86-0431-85383815

E-mail: dongxiangting888@163.com (Xiangting Dong)

**Supplementary Information**

**Manufacture process and utilization of specially designed spinneret**

The manufacturing process of specially designed spinneret is depicted in Fig. S1. Three 12 # stainless steel needles (inner/outer diameter: 0.90/1.26 mm) were truncated into 6 cm in length, and two of them were bended at a certain angle and assembled on either side of the other one with a piece of double sided sticky tape. A piece of copper wire used as electrode was then winded onto the three stainless steel needles. A plastic nozzle was cut to an appropriate length and put on over the tip of the three stainless steel needles. At last, an all-purpose adhesive was used to seal the space between the stainless steel needles and the cut section of plastic nozzle.


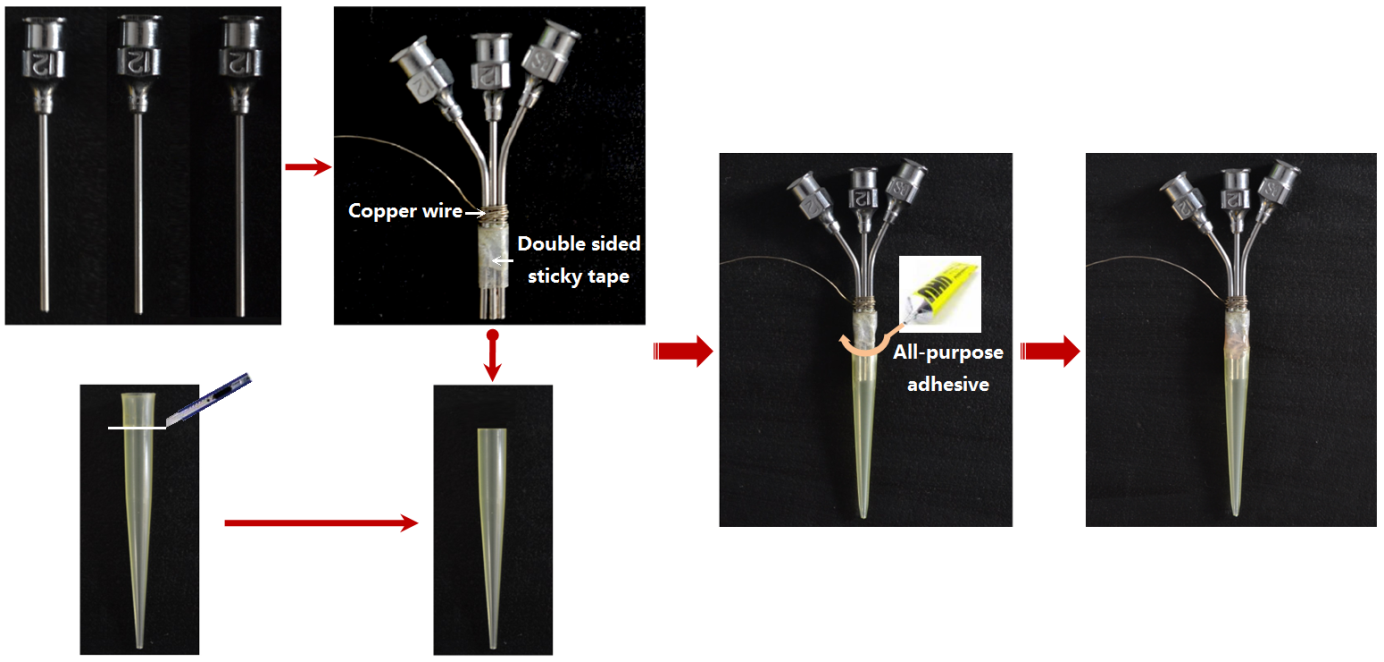


**Figure S1:** Schematic diagram for manufacturing process of specially designed spinneret.

Fig. S2 shows a digital photo of the initial microribbon formation process. One can see that the three spinning solutions form stable and clear interfaces in the spinneret. Owing to high viscosity of the spinning solutions, the three spinning solutions diffuse and mix very slowly. As the electrospinning speed is fast, and the spinneret is short, the three spinning solutions jet out before they mix together. By using the specially designed spinneret, tricolor flag-liked microribbons possessing same structure can be fabricated, as indicated in Fig. S3.


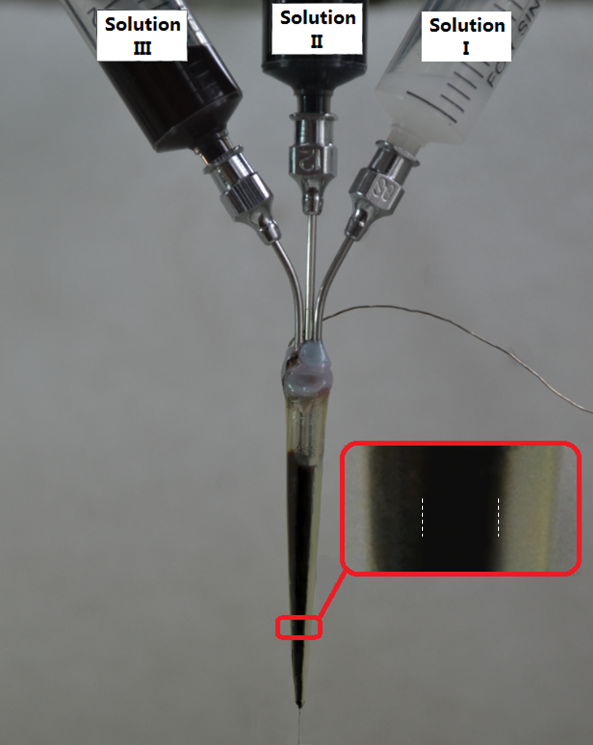


**Figure S2:** Digital photo of the initial microribbon formation process.


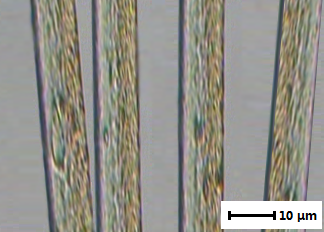


**Figure S3:** Optical microscope photograph of tricolor flag-liked microribbons.

**Preparation process, morphology, phase and magnetism of Fe_3_O_4_ nanoparticles**

One typical synthetic procedure was as follows: 5.4060 g of FeCl_3_·6H_2_O, 2.7800 g of FeSO_4_·7H_2_O, 4.0400 g of NH_4_NO_3_, and 1.9000 g of PEG were added to 100 mL of deionized water to form a uniform solution under vigorous stirring at 50 °C. To prevent the oxidation of Fe^2+^, the reactive mixture was kept under an argon atmosphere. After the mixture had been bubbled with argon for 30 min, 0.1 mol L^-1^ of NH_3_·H_2_O was dropwise added into the mixture until the pH value was above 11. Then the system was continuously bubbled with argon for 20 min at 50 °C, and black precipitates were formed. The precipitates were collected from the solution by magnetic separation, washed with deionized water for three times, and then dried in an electric vacuum oven for 12 h at 60 °C. The obtained Fe_3_O_4_ nanoparticles are spherical in shape (Fig. S4a), and the particle size is 11.95±1.47 nm (Fig. S4b). The XRD patterns (Fig. S4c) of the as-prepared Fe_3_O_4_ nanoparticles conform to the cubic structure of Fe_3_O_4_ (PDF 74-0748). The XRD analysis results of tricolor flag-liked microribbons array demonstrate that tricolor flag-liked microribbons array contains Fe_3_O_4_, and the broad diffraction peak extending from 15 ° to 20 ° is attributed to the amorphous PMMA and PANI. The saturation magnetization of the Fe_3_O_4_ nanoparticles before coating by oleic acid is 62.02 emu g^-1^, and the remanence nears zero (Fig. S4d).


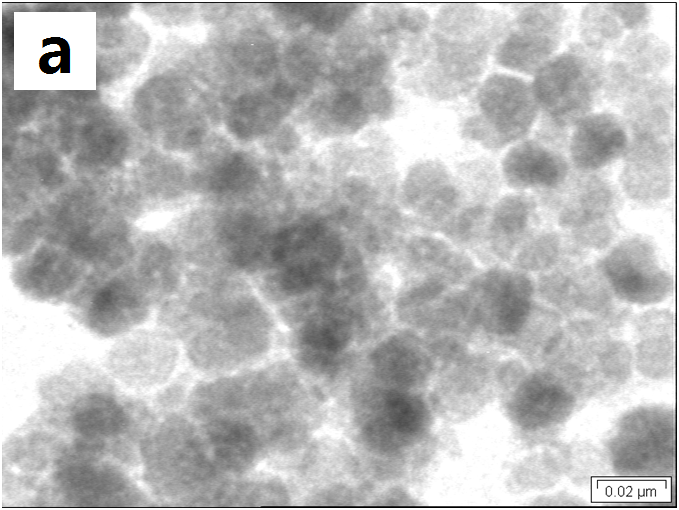


**Figure S4:** (**a**) TEM image and (**b**) histogram of particle size of Fe_3_O_4_ nanoparticles. (**c**) XRD patterns of Fe_3_O_4_ nanoparticles and tricolor flag-liked microribbons array. (**d**) Hysteresis loop for Fe_3_O_4_ nanoparticles.

**Impact of different amounts of ANI, CSA and APS on conductivity of conductive region**

For performing the electrical conductivity tests, 2 mL of the spinning solution II was dripped onto a piece of glass and the solvent was allowed to volatilize at room temperature for 48 h. The dosages of ANI, CSA, APS and the corresponding conductivities are shown in Table S1. One can see that the conductivity reaches up to the order of 10^-2^ S cm^-1^, and it increases not much with adding more ANI than 0.1500 g. Thus the spinning solution II containing 0.1500 g ANI was adopted for this study.

**Table S1:** Dosages of ANI, CSA, APS and corresponding conductivities

| ANI (g) | CSA (g) | APS (g) | Conductivity (S cm^-1^) |
| --- | --- | --- | --- |
| 0.0500 | 0.0625 | 0.1225 | 5.650×10^-6^ |
| 0.1000 | 0.1249 | 0.2450 | 6.342×10^-4^ |
| 0.1500 | 0.1873 | 0.3676 | 2.425×10^-2^ |
| 0.2000 | 0.2497 | 0.4900 | 4.608×10^-2^ |
| 0.2500 | 0.3125 | 0.6125 | 5.939×10^-2^ |
